# Surgical outcomes of acute type A aortic dissection in dialysis patients: lessons learned from a single-center’s experience

**DOI:** 10.1038/s41598-022-09448-7

**Published:** 2022-03-30

**Authors:** Zhigang Wang, Pingping Ge, Lichong Lu, Min Ge, Cheng Chen, Lifang Zhang, Dongjin Wang

**Affiliations:** 1 Department of Cardio-Thoracic Surgery, Affiliated Drum Tower Hospital, Medical School of Nanjing University, Zhongshan Road 321, Nanjing, 210008 China; 2 Department of General Practice, Nanjing First Hospital, Nanjing Medical University, Nanjing, China; 3 Department of Psychiatry, The First Affiliated Hospital, Zhengzhou University, Zhengzhou, China

**Keywords:** Cardiology, Nephrology

## Abstract

There is a paucity of data describing the safety and efficacy of acute type A aortic dissection (ATAAD) repair surgeries in dialysis patients. Our study aimed to investigated the influence of dialysis on early and late outcomes in end-stage renal disease (ESRD) patients who received repair surgery for ATAAD. A total of 882 ATAAD patients who received emergency aortic dissection repair at our center from January 2015 to December 2019 were retrospectively screened in this study and divided into the dialysis group (n = 16) and the non-dialysis group (n = 866), depending on whether they required dialysis for preoperative ESRD. No significant difference of age, preoperative hemodynamics, organ ischemia conditions, operative variables as well as the 30-Day mortality and in-hospital complications was discovered between two groups. However, the survival rates and the proportion of late aortic event (sudden death and reoperation) free population at 1 and 3 years after surgery were significantly decreased in dialysis patients compared to non-dialysis patients. Our study indicated that the short-term surgical outcomes of ATAAD in dialysis patients were comparable to non-dialysis patient. However, the dialysis patients were associated with a worse long-term prognosis.

## Introduction

It has been well studied that patients with end-stage renal disease (ESRD) associate with decreased life expectancy and are more vulnerable to develop cardiovascular events compared to healthy individuals^1–3^. As a well-established treatment method, increasing ESRD patients are receiving regular dialysis treatment. In 2010, the incidence and prevalence of patients who require hemodialysis were 147.3/million and 509/million in Beijing^4^.

Acute type A aortic dissection (ATAAD) is a critical disease that often associates with lethal outcomes. It often progresses rapidly and develops life-threatening complications, such as aortic rupture and cardiac tamponade^5^. Although outcomes of ATAAD have been improving in recent years due to the advance of techniques^6^, it is still a dangerous condition, especially in patients with other comorbidities like ESRD. The hemodynamic and electrolytes homeostasis is often disturbed under dialysis which posts additional challenges for the surgical repair of ATAAD^7^. However, the outcomes of such patients had not been well described. In this study we described both the short- and long-term outcomes of dialysis patients who received ATAAD repair surgery.

## Methods and materials

A total of 882 consecutive patients who received emergent ATAAD surgery at Nanjing Drum Tower Hospital between January 2015 and December 2019 were retrospective screened for this study. The diagnosis of ATAAD was made on the basis of enhanced computed tomography and the acute ATAAD was characterized as patients within 14 days of symptom onset. Among all 882 patients, 16 patients were receiving hemodialysis or peritoneal dialysis therapy for ESRD before the onset of aortic dissection. No patients received renal transplantation before the onset of the ATAAD.

The 882 patients were divided into two groups according to whether they were receiving dialysis therapy before the surgery (dialysis group, n = 16; non-dialysis group, n = 866). Patients’ medical records and imaging results were reviewed. The institutional review board of the Nanjing Drum Tower Hospital approved this study (No. BL2014004) and waived the requirement for informed consent because of the retrospective nature of the study. The study was conducted in accordance with the Declaration of Helsinki (as revised in 2013).

The Follow-up was accomplished by telephone interview with the patient, family members, or the patient’s referring physicians from December 2015 to December 2020. Late aortic events were defined as residual aneurysm or anastomotic pseudoaneurysm, that requires another surgical repair, fatal aortic rupture, sudden death, and expansion of more than 6 cm in diameter of a residual aneurysm^8^.

### Surgical procedure and postoperative treatment

The ATAAD repair surgery were carried out as described previously^9^. Briefly, surgical procedures including routine median sternotomy, cardiopulmonary bypass, and intermittent cardioplegic arrest with hypothermic circulatory arrest were conducted similarly between two groups. Appropriate distal surgical method was chosen depending on the location of the intimal tear and the extent of dissection. For the proximal segment, a root reinforcement reconstruction was routinely performed. The aortic valve replacement or Bentall procedure was performed when the dissection involved the coronary ostia or aortic valve, or was in the presence of an aortic root aneurysm.

After the operation, all patients were transferred to the intensive care unit (ICU). Continuous renal replacement therapy was started for dialysis patients 6 h after the operation.

### Statistical analyses

Continuous variables were expressed as mean ± standard deviations or median with interquartile and were analyzed by Student’s *t*-test or the Mann–Whitney *U* test. Categorical variables were presented as n (%) and analyzed with the chi-square or Fisher’s exact test.

A systematic literature review was conducted and identified potential predictors such as age, sex, cause, medical history, and operative procedures as predictors for prognosis in ATAAD. To reduce the influence of these confounding baseline parameters, a one-to-one propensity score matching method was applied to analyze the short-term outcomes (calipers of width 0.02 standard deviations of the logit of the propensity score), baseline characteristics and variables of interest that associated with outcomes (variables excepting for laboratory data listed in Table 1 and intro-operative variables listed in Table 3) were included in the analysis. Cumulative survival and late aortic event free rate were calculated by the Kaplan–Meier method which was performed using STATA, version 15.0 (Stata Corporation, College Station, TX), and the difference was determined by the long-rank test. The rest of statistical analyses were carried out using IBM SPSS Version 25.0 (SPSS Science, Chicago, IL, USA). A two-sided *p*-value < 0.05 was considered statistically significant.

**Table 1:** Comparison of preoperative variables.

Variables	Total (n = 882)	Overall cohort			PSM cohort		
		Non-dialysis (n = 866)	Dialysis (n = 16)	*P* Value	Non-dialysis (n = 16)	Dialysis (n = 16)	*P* Value
DeBakey type I (%)	727 (82.4)	715 (82.6)	12 (75.0)	0.503	13 (81.3)	12 (75.0)	1.000
**Demographic data**							
Age (year)	53.0 ± 13.2	53.1 ± 13.2	47.1 ± 11.2	0.069	54.2 ± 10.5	47.1 ± 11.2	0.073
Male (%)	646 (73.2)	638 (73.7)	8 (50.0)	0.045	8 (50.0)	8 (50.0)	1.000
Obesity (BMI > 30 kg/m^2^) (%)	99 (14.1)	99 (14.4)	0 (0)	0.147	2 (18.2)	0 (0)	0.157
**Medical history**							
Hypertension (%)	639 (72.4)	623 (71.9)	16 (100)	0.009	16 (100)	16 (100)	–
Diabetes mellitus (%)	20 (2.3)	20 (2.3)	0 (0)	1.000	0 (0)	0 (0)	–
Previous cardiovascular disease (%)	28 (3.2)	28 (3.2)	0 (0)	1.000	1 (6.3)	0 (0)	1.000
Cerebrovascular disease (%)	33 (3.8)	32 (3.7)	1 (9.1)	0.346	1 (6.3)	1 (9.1)	1.000
Marfan syndrome (%)	24 (2.7)	24 (2.8)	0 (0)	1.000	2 (12.5)	0 (0)	0.484
**Previous cardiac surgery (%)**							
PCI (%)	9 (1.0)	9 (1.0)	0 (0)	1.000	1 (6.3)	0 (0)	1.000
TEVAR (%)	18 (2.1)	18 (2.1)	0 (0)	1.000	2 (12.5)	0 (0)	0.499
CABG (%)	1 (0.1)	1 (0.1)	0 (0)	1.000	0 (0)	0 (0)	–
AVR (%)	14 (1.6)	14 (1.6)	0 (0)	1.000	1 (6.3)	0 (0)	1.000
Limb ischemia (%)	107 (12.1)	107 (12.4)	0 (0)	0.242	3 (18.8)	0 (0)	0.226
Mesenteric ischemia (%)	34 (3.9)	34 (3.9)	0 (0)	1.000	1 (6.3)	0 (0)	1.000
Cerebral ischemia (%)	78 (8.8)	77 (8.9)	1 (6.3)	1.000	4 (25.0)	1 (6.3)	0.333
Coronary ischemia (%)	46 (5.2)	46 (5.3)	0 (0)	1.000	3 (18.8)	0 (0)	0.226
**Location of the entry tear**							
Ascending aorta (%)	556 (63.0)	549 (63.4)	7 (43.8)	0.107	8 (50.0)	7 (43.8)	0.723
Aortic arch (%)	117 (13.3)	113 (13.0)	4 (25.0)	0.252	3 (18.8)	4 (25.0)	1.000
Descending aorta or unknown (%)	209 (23.7)	204 (23.6)	5 (31.3)	0.552	4 (25.0)	5 (31.3)	1.000
Hypotension (%)	26 (2.9)	24 (2.8)	2 (12.5)	0.078	3 (18.8)	2 (12.5)	1.000
Pericardial tamponade (%)	151 (17.1)	147 (17.0)	4 (25.0)	0.498	0 (0)	4 (25.0)	0.101
**Preoperative laboratory data**							
WBC (10^9^/L)	11.0 (8.3, 14.1)	11.1 (8.4, 14.1)	7.7 (6.3, 9.7)	0.014	14.0 ± 6.3	8.6 ± 3.0	0.008
Haemoglobin (g/L)	123.5 ± 29.2	124.1 ± 29.0	88.3 ± 14.7	< 0.001	111.6 ± 26.3	88.3 ± 14.7	0.012
PLT (10^9^/L)	144.0 (108.0, 184.0)	144.0 (108.0, 184.0)	141.5 (123.8, 177.3)	0.899	113.1 ± 56.3	149.6 ± 43.0	0.067
Fibrinogen (g/L)	2.5 ± 1.4	2.5 ± 1.4	2.9 ± 1.1	0.140	2.2 ± 0.9	2.9 ± 1.1	0.077
Triglyceride (mmol/L)	1.0 (0.7, 1.5)	1.0 (0.7, 1.5)	1.4 (0.6, 1.6)	0.639	0.8 (0.5, 1.3)	1.4 (0.6, 1.6)	0.417
CRP (mg/dl)	19.2 (4.6, 75.7)	19.2 (4.6, 76.4)	28.2 (4.9, 54.7)	0.902	29.9 (7.4, 71.0)	28.2 (4.9, 54.7)	0.461
D-dimer (ng/mL)	4.7 (2.3, 9.4)	4.6 (2.3, 9.4)	5.9 (4.5, 10.4)	0.076	7.1 (2.8, 22.8)	5.9 (4.5, 10.4)	0.661
Albumin (g/L)	37.3 (33.8, 40.1)	37.3 (33.8, 40.1)	34.1 (31.0, 38.0)	0.134	34.6 ± 5.3	34.8 ± 4.7	0.934
TnT (ng/ml)	0.02 (0.01, 0.14)	0.02 (0.01, 0.14)	0.06 (0.03, 0.12)	0.046	0.08 (0.04, 0.21)	0.06 (0.03, 0.12)	0.568
ALT (U/L)	25.6 (15.7, 46.9)	25.6 (15.8, 47.0)	15.2 (9.9, 53.5)	0.185	49.3 (18.4, 194.5)	15.2 (9.9, 53.5)	0.062
Bun (mmol/L)	7.2 (5.6, 9.5)	7.1 (5.5, 9.4)	18.3 (13.5, 28.4)	< 0.001	10.7 ± 2.8	20.8 ± 9.5	< 0.001
sCr (mg/dl)	112.7 ± 129.3	99.2 ± 70.3	841.8 ± 345.1	< 0.001	160.6 (115.5, 203.1)	786.9 (585.8, 988.9)	< 0.001
eGFR (ml/min)	85.6 ± 43.0	87.8 ± 41.6	9.4 ± 5.6	< 0.001	80.3 ± 20.5	9.4 ± 5.6	< 0.001
Total bilirubin (mg/dl)	15.3 (10.8, 22.6)	15.3 (10.9, 22.6)	7.0 (4.9, 16.2)	0.002	18.5 (11.9, 29.7)	7.0 (4.9, 16.2)	0.004
INR	1.1 (1.0, 1.2)	1.1 (1.0, 1.2)	1.3 (1.1, 1.6)	0.011	1.2 (1.1, 1.3)	1.3 (1.1, 1.6)	0.415
APTT (s)	28.9 (26.2, 35.2)	28.9 (26.2, 35.1)	29.3 (25.4, 42.3)	0.568	30.1 (27.0, 38.6)	29.3 (25.4, 42.3)	0.968

Values for categorial variables are given as count (percentage); values for continuous variables are given as median (interquartile range) or mean ± standard deviation.

*BMI* body mass index, *WBC* white blood cell, *Bun* blood urea nitrogen, *sCr* serum creatinine, *PLT* platelet, *ALB* albumin, *CRP* c-reactive protein, *eGFR* estimated glomerular filtration rate, *INR* international normalized ratio, *PSM* propensity score matching.

### Ethics declarations

All procedures performed in studies involving human participants were in accordance with the ethical standards of Nanjing Drum Tower Hospital of Medicine Ethics Committee for Clinical studies at which the studies were conducted (Approval number: No. BL2014004). Written informed consent was waived due to the nature of the study.

## Results

Patients’ preoperative parameters and anatomical characteristics of the ATAAD lesions were shown in Table 1. Our data showed that the average age of patients in the dialysis group was similar to those in the non-dialysis group (47.1 ± 11.2 years vs. 53.1 ± 13.2 years, *p* > 0.05). However, significantly more patients in the dialysis group had hypertension histories (*p* = 0.009). Interestingly, no significant difference was identified in preoperative hemodynamic measurements and organ malperfusion conditions between the two groups. On the other hand, the levels of leukocyte count, haemoglobin, creatinine, and blood urea nitrogen were significantly different between the two groups. In addition, we found out a clear trend that dialysis patients were more likely to have primary entry tear in the aortic arch, even though the difference was not statistically significant.

As shown in Table 2, the leading primary cause for ESRD were hypertension (n = 10) followed by chronic glomerulonephritis (n = 5). The mean duration of dialysis history before the onset of ATAAD was 4.5 ± 3.5 years, and 87.5% of these patients were receiving hemodialysis (rather than peritoneal dialysis).

**Table 2:** Characteristics of the renal disease.

	Total (n = 16)
**Primary cause of end-stage renal disease**	
Hypertension	10 (62.5%)
Chronic glomerulonephritis	5 (31.3%)
Unknown	1 (6.3%)
**Type of dialysis**	
Hemodialysis	14 (87.5%)
Peritoneal	2 (12.5%)
**Type of blood access**	
Upper limb hemodialysis shunt	13 (81.3%)
Superficialization of the brachial artery	1 (6.3%)
Duration of dialysis (years)	4.5 ± 3.5

Values for categorial variables are given as count (percentage); values for continuous variables are given as mean ± standard deviation.

As shown in Table 3, operative variables like arterial cannulation sites, aortic arch surgery methods, distal surgical techniques, and cardiopulmonary bypass duration were similar between the two groups. However, dialysis patients were more likely to receive root construction, compared to patients in the non-dialysis group.

**Table 3:** Comparison of operative variables.

Variables	Total (n = 882)	Overall cohort			PSM Cohort		
		Non-dialysis (n = 866)	Dialysis (n = 16)	*P* Value	Non-dialysis (n = 16)	Dialysis (n = 16)	*P* Value
**Intro-operative variables**							
CABG (%)	51 (5.8)	50 (5.8)	1 (6.3)	1.000	2 (12.5)	1 (6.3)	1.000
CPB time (min)	232.3 ± 67.9	232.3 ± 67.6	235.8 ± 85.1	0.984	272.3 ± 95.3	235.8 ± 85.1	0.291
Aortic cross-clamp time (min)	154.0 (124.0, 194.0)	154.0 (124.0, 194.0)	138.0 (106.8, 194.0)	0.374	171.5 ± 60.6	156.0 ± 63.9	0.366
DHCA time (min)	29.5 ± 12.5	29.5 ± 12.5	28.2 ± 11.9	0.710	24.3 ± 12.2	28.2 ± 11.9	0.335
**Cannulation**							
Axillary artery (%)	171 (19.4)	166 (19.2)	5 (31.3)	0.213	6 (37.5)	5 (31.3)	0.710
Femoral artery (%)	230 (26.1)	225 (26.0)	5 (31.3)	0.578	5 (31.3)	5 (31.3)	1.000
Axillary + femoral artery (%)	442 (50.1)	436 (50.3)	6 (37.5)	0.309	5 (31.3)	6 (37.5)	1.000
**Root procedure**							
Bentall (%)	202 (22.9)	201 (23.2)	1 (6.3)	0.139	6 (37.5)	1 (6.3)	0.083
Root reconstruction (%)	641 (72.7)	626 (72.3)	15 (93.8)	0.085	10 (62.5)	15 (93.8)	0.083
Valve sparing root replacement (%)	35 (4.0)	35 (4.0)	0 (0)	1.000	3 (18.8)	0 (0)	0.248
**Distal surgical technique**							
Hemi-arch replacement (%)	179 (20.3)	175 (20.2)	4 (25.0)	0.546	7 (43.8)	4 (25.0)	0.264
Total arch + frozen elephant trunk (%)	422 (47.8)	415 (47.9)	7 (43.8)	0.741	10 (62.5)	7 (43.8)	0.288
Arch fenestrated stent graft (%)	268 (30.4)	263 (30.4)	5 (31.3)	1.000	10 (62.5)	5 (31.3)	0.288

Values for categorial variables are given as count (percentage); values for continuous variables are given as median (interquartile range) or mean ± standard deviation.

*MVR* mitral valve replacement, *MVP* mitral valvuloplasty, *TVP* tricuspid valvuloplasty, *CABG* coronary artery bypass graft, *CPB* cardiopulmonary bypass, *DHCA* deep hypothermic circulatory arrest, *PSM* propensity score matching.

Next, we examined the early prognosis of ATAAD repair surgery in both groups (Table 4). Before propensity score matching, the 30-Day mortality rate of patients in the dialysis group was similar to the non-dialysis group (12.5% vs. 11.4%, *p* > 0.05). In the dialysis group, the causes for 30-Day in-hospital death were intracranial hemorrhage (n = 1) and multi-organ failure (n = 1). Interestingly, the ICU and hospital stay were similar between the two groups as well as operation associated complications. Meanwhile, the drainage volume 24 h after surgery, mechanical ventilation duration, and re-intubation rate were significantly increased in the dialysis group (*p* < 0.05). After propensity score matching, the 30-Day mortality remained similar between the two groups. In addition, the differences of other postoperative parameters were no longer identifiable between the two groups after propensity score matching.

**Table 4:** Comparison of postoperative variables.

Variables	Total (n = 882)	Overall cohort			PSM Cohort		
		Non-dialysis (n = 866)	Dialysis (n = 16)	*P* Value	Non-dialysis (n = 16)	Dialysis (n = 16)	*P* Value
**Postoperative complications (%)**							
Re-exploration for bleeding (%)	33 (3.7)	33 (3.8)	0 (0)	1.000	4 (25.0)	0 (0)	0.101
Dialysis (%)	148 (16.8)	132 (15.2)	16 (100.0)	< 0.001	8 (50.0)	16 (100.0)	0.002
Stroke (%)	69 (7.8)	68 (7.9)	1 (6.3)	1.000	0 (0)	1 (6.3)	1.000
Paraplegia (%)	29 (3.3)	28 (3.2)	1 (6.3)	0.417	0 (0)	1 (6.3)	1.000
Re-intubation (%)	37 (4.2)	34 (3.9)	3 (18.8)	0.026	2 (12.5)	3 (18.8)	1.000
Tracheostomy (%)	36 (4.1)	35 (4.0)	1 (6.3)	0.490	1 (6.3)	1 (6.3)	1.000
Deep sternal wound infection (%)	13 (1.5)	12 (1.4)	1 (6.3)	0.213	1 (6.3)	1 (6.3)	1.000
Sepsis (%)	8 (0.9)	8 (0.9)	0 (0)	1.000	1 (6.3)	0 (0)	1.000
Intracranial hemorrhage (%)	6 (0.7)	5 (0.6)	1 (6.3)	0.104	1 (6.3)	1 (6.3)	1.000
Gastrointestinal bleeding (%)	4 (0.5)	4 (0.5)	0 (0)	1.000	1 (6.3)	0 (0)	1.000
Drainage volume 24 h after surgery (ml)	520.0 (300.0, 869.5)	510.0 (300.0, 864.5)	680.0 (602.5, 1042.5)	0.033	520.0 (345.0, 835.0)	680.0 (602.5, 1042.5)	0.051
Ventilation time (hour)	17.0 (11.0, 43.0)	17.0 (11.0, 43.0)	33.0 (14.6, 60.6)	0.046	61.5 (16.8, 146.8)	33.0 (14.6, 60.6)	0.269
ICU Stay time (day)	4.0 (3.0, 7.0)	4.0 (3.0, 7.0)	6.5 (4.3, 9.0)	0.083	8.0 (6.0, 12.0)	6.5 (4.3, 9.0)	0.196
Hospital stay time (day)	20.9 ± 12.1	21.0 ± 12.2	18.4 ± 11.5	0.279	27.5 (10.8, 35.8)	17.5 (9.3, 21.3)	0.086
30-Day mortality (%)	101 (11.5)	99 (11.4)	2 (12.5)	0.704	2 (12.5)	2 (12.5)	1.000

Values for categorial variables are given as count (percentage); values for continuous variables are given as median (interquartile range) or mean ± standard deviation.

*ICU* intensive care unit, *PSM* propensity score matching.

**Figure 1:**
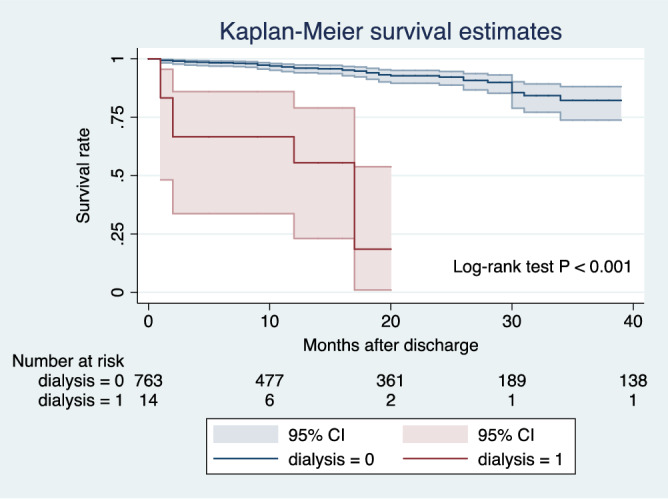
Kaplan–Meier curves for overall cumulative survival of dialysis and non-dialysis patients suffering from acute type A aortic dissection.

105 patients (11.9%) died during the hospitalization period. The median follow-up was 29 months. 46 patients (5.9%) who were lost to follow-up and 1 patient who committed suicide 6 months after hospital discharge were identified as censored data. A total of 43 patients in the non-dialysis group and 5 patients in the dialysis group died during the follow-up period (Fig. 1). The 1-year and 3-year survival rates was significantly decreased in dialysis patients compared to non-dialysis patients (59.3 ± 14.3% vs. 96.8 ± 0.7% and 29.7 ± 16.5% vs. 90.1 ± 1.7%, respectively *p* < 0.001, log rank).

**Figure 2:**
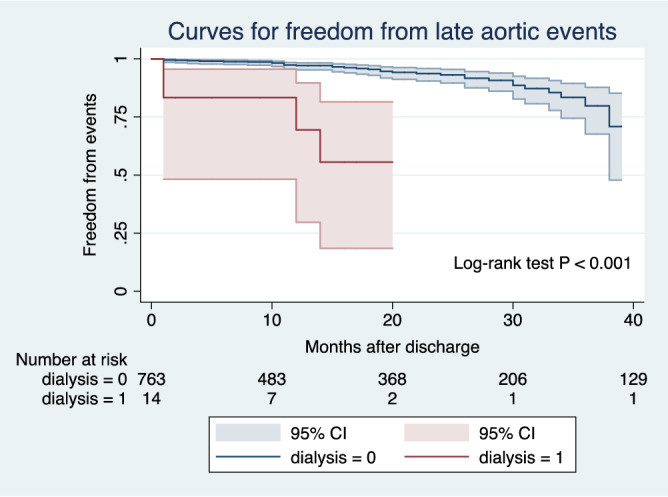
Kaplan–Meier curves for freedom from late aortic events of dialysis and non-dialysis patients suffering from acute type A aortic dissection.

Late aortic events including sudden death (n = 3) and reoperation at a different site of the aorta (n = 1) were identified in the dialysis group. On the other hand, fatal aortic rupture (n = 9), sudden death (n = 12) and reoperation at a different site of the aorta (n = 21) were identified in the non-dialysis group. As shown in the Fig. 2, the late aortic event free survival was significantly decreased in dialysis patients compared to non-dialysis patients at both 1 and 3 years after the operation (72.5 ± 14.1% vs. 97.7 ± 0.6% and 60.4 ± 14.1% vs. 90.6 ± 1.8%, respectively *p* < 0.001).

## Discussion

In this study, our data indicated that the preoperative parameters, including hemodynamics and organ malperfusion conditions, were similar between patients without or without dialysis. Furthermore, no significant difference of postoperative parameters as well as other short-term prognosis measurements was identified in dialysis patients after propensity score matching. However, the long-term mortality and incidence of late aortic events was significantly increased in dialysis patients compared to non-dialysis patients.

Our study indicated that only 1.8% (16/882) of all ATAAD patients were receiving dialysis treatment due to ESRD, which was similar to the 1–3% prevalence identified in other previous studies^7,10,11^. However, the treatment for this subgroup of patients is difficult and often associates with higher morbidity, such as cerebrovascular diseases, hypertension, and diabetes^12^.

A multi-center registry study conducted in Germany reported that the incidence of primary entry tear in aortic arch was 14.5%^13^, which was similar to the 13% (113/866) we identified in our study among non-dialysis patients. In contrast, 25% (4/16) of the dialysis patients developed a primary entry tear in aortic arch. This difference might due to the increased calcification in the aortic arch area during dialysis treatment^7^, which might explain why intimal tears are more likely to occur in the atherosclerotic aortic arch as well.

Conflicting studies had been published about the safety of surgical repair in dialysis patients. Some previous studies identified ESRD as a major risk factor for postoperative morbidity and mortality. Liu and colleagues^14^ reported that the adjusted mortality rate in dialysis-dependent patients was 3-times higher compared to those with normal renal function. In addition, Okada et al. identified that the severe renal dysfunction was an independent risk factor for in-hospital death in non-dialysis patients^15^. On the contrary, another retrospective study which included 960 patients suggested that although the in-hospital mortality rate was increased in dialysis patients (16% vs*.* 6%), no statistically difference was achieved^7^. Similarly, no significant difference of 30-Day mortality rate was identified in our cohort between dialysis patients and non-dialysis patients. Furthermore, our results also seemed contradicting to some previous studies which suggested that the ESRD was associated with increasing postoperative complications^16,17^. We hypothesized the relatively improved short-term prognosis we observed in this study was due to the improvement of surgical as well as critical care techniques and more careful matching of baseline characteristics.

A dilated aorta is believed to be associated with worse long-term prognosis of ATAAD. However, our clinical experience suggests that extended aortic replacement can be especially dangerous for dialysis patients due to the increased operative invasiveness and prolonged operation time. Therefore, we suggest that the extended aortic replacement in dialysis patients should be avoided unless the clear identification of expanded lesions on imaging results.

It has been well known in the field that the control of hypertension is critical to manage patients with residual aneurysms^18^. All dialysis patients in our study had hypertension, compared to the 70% identified among non-dialysis patients. Previous studies have shown the efficacy of beta-blocking agents on prevention of aortic dissection and dilatation in Marfan syndrome patients^19^. Similarly, our previous study also suggested that regular beta-blockers treatment after discharge was associated with decreased long-term mortality in ATAAD patients who received aortic dissection repair surgery^20^. Considering the fact that the renin-angiotensin system was more activated in ESRD due to the hemodynamic changes, beta-blockers seemed to be more beneficial in such group of patients. For dialysis patients with hypertension, strong consideration should be given to the prescription of beta-blockers after aortic dissection repair surgery.

In addition, one of our previous studies showed that the concomitant hypertension identified upon hospital administration was an independent risk factor for long-term mortality in ATAAD patients^20^. These observations indicated that strict medication adherence as well as blood pressure control after discharge is critical in the management of patients who received aortic dissection surgery repair, especially for dialysis patients.

In summary, these results indicated that the ATAAD repair surgery was relatively safe in dialysis patients but closer follow-up should be planned.

## Limitations

This study had some limitations. Firstly, this was a retrospective study conducted in a single center with limited dialysis patients. A multi-center study with a larger cohort is needed to validate our findings in future. Secondly, our surgical technique had evolved over the study period which might influence the results. Finally, the result of this study should be interpreted with caution due to limited follow-up period and incomplete demographic data from some patients.

## Conclusion

Our study indicated that the short-term outcomes for dialysis patients who received conventional ATAAD repair surgery were acceptable. However, these patients were associated with a worse long-term prognosis. These results reemphasized the need for close follow-up examination and precautions should be made for late aortic events in dialysis patients who received ATAAD repair surgery. Further prospective multicenter studies aim to identify approaches to reduce late complications are required.
